# Artemisinin-based combination therapy availability and use in the private sector of five AMFm phase 1 countries

**DOI:** 10.1186/1475-2875-12-135

**Published:** 2013-04-22

**Authors:** Ben Davis, Joel Ladner, Kelley Sams, Ebru Tekinturhan, Donald de Korte, Joseph Saba

**Affiliations:** 1 Axios International, 7, Boulevard de la Madeleine, Paris, 75001, France; 2 Epidemiology and Public Health Department, Hôpital Charles Nicolle, Rouen University Hospital, 1, rue de Germont, Rouen cedex, 76031, France; 3 Department of Anthropology, University of South Florida, 4202 E. Fowler Avenue, SOC 107, Tampa, FL, 33620-8100, USA; 4 Malaria Initiative, Novartis Pharma AG, Novartis Campus, WSJ 210.10.21, Basel, 4056, Switzerland

**Keywords:** Malaria treatment, AMFm, ACT, Global fund, Private

## Abstract

**Background:**

In 2009, the Global Fund to Fight AIDS, Tuberculosis and Malaria established the Affordable Medicines Facility-malaria (AMFm) in order to increase access to quality-assured artemisinin combination therapy (QAACT). AMFm Phase 1, which includes nine pilot programmes in eight countries, was launched in 2009. The objective of this study was to assess anti-malarial stock and purchase patterns at private outlets in five AMFm Phase 1 countries in regard to three of the core AMFm goals: increase the affordability of QAACT, increase the availability of QAACT, and crowd out artemisinin monotherapies and other substandard therapies.

**Methods:**

The study was conducted between April and May 2012 and included interviews with personnel in 598 private pharmaceutical outlets in Ghana, Kenya, Nigeria, Tanzania, and Uganda. Questionnaires were administered at private retail outlets and the data were analyzed to assess within- and between-country differences in QAACT price, availability, and popularity.

**Results:**

AMFm medications were less expensive than their non-AMFm counterparts, yet prices for both types were above country-specific suggested retail prices. Market penetration of AMFm QAACT in both urban and rural areas was high, although stock-outs of both AMFm and non-AMFm products were more common in rural compared with urban outlets in Ghana and Kenya (p = 0.0013). Government recommendation was the most significant factor influencing anti-malarial stock choices in urban (41.5%) and rural (31.9%) outlets. The three top-selling anti-malarials reported for both urban and rural areas in each country were, with the exception of rural Uganda and urban Nigeria, combination therapies.

**Conclusions:**

Results from this study indicate that the AMFm has not fully achieved its affordability and crowd-out objectives. Still, the final purchase price of AMFm QAACT was substantially lower than non-AMFm equivalents. Moreover, for both urban and rural areas, AMFm QAACT availability was found to be high, and the various forms of QAACT were the best-selling products among all anti-malarials. These findings suggest a continued need for initiatives like the AMFm that improve the affordability and accessibility of QAACT. Similar programmes may be especially effective if employed in combination with rapid diagnostic testing to ensure the appropriate use of these products.

## Background

It is estimated that there were 216 million cases of malaria and 655,000 malaria deaths in 2010. The majority of cases (81%) and deaths (91%) occurred in the World Health Organization (WHO) African region. In 2010, there were 3.3 billion people at risk for malaria worldwide, of whom 1.2 billion (47%) were considered at high risk (> 1 case/1,000 population) and living in the WHO African Region [1].

In 2006, the WHO recommended the first-line use of artemisinin-based combination therapy (ACT) to address the resistance of *Plasmodium falciparum* to monotherapies and improve treatment outcomes [2]. Since 2000, the use of ACT has helped reduce malaria-related deaths in Europe by 99%, the Americas by 55%, and the Western Pacific by 42%. During the same period, however, malaria mortality in Africa was reduced by only 33% [1]. In 2010, the WHO updated its malaria treatment guidelines to recommend that ACT replace oral artemisinin-based monotherapy [3]. As of November 2011, there were still 25 countries, primarily located in the Africa Region, which continued to allow the marketing of artemisinin monotherapy [1].

The private sector plays a key role in the treatment of malaria. A study of global malaria treatment found that up to 60% of malaria medications are delivered in the private sector [4]. There are several studies suggesting that achieving the Roll Back Malaria Partnership goal of providing 80% of malaria patients with effective treatment within 24 hours from symptom onset will depend on increasing the availability of anti-malarial drugs outside the public sector [5-7]. This finding is supported by an analysis of six African countries, which found that the majority of treatment-seeking febrile patients in the Democratic Republic of Congo, Madagascar, Nigeria, and Uganda initially sought treatment in the private sector [8]. Additionally, the private sector was the source of more than half of the anti-malarials acquired for children under the age of five with fever in these four countries [8].

In order to address the need for increased access to ACT in both the private and public sector, the Global Fund to Fight AIDS, Tuberculosis and Malaria established the Affordable Medicines Facility-malaria (AMFm). This programme negotiates reduced pricing with ACT manufacturers that provide quality-assured ACT (QAACT) by agreeing to pay a significant co-payment, a strategy which is intended to reduce the final purchase price of ACT for patients. As defined by AMFm, QAACT is a formulation of ACT that has been approved by the WHO Prequalification Programme, which assures the safety, quality, and efficacy of medicinal products [9]. The four core objectives of the AMFm are to (1) increase the affordability of QAACT; (2) increase the availability of QAACT; (3) increase the use of QAACT; and (4) crowd out monotherapies and other substandard therapies [10]. Substandard therapies are those that either do not ensure high cure rates of *P. falciparum* malaria or increase the possibility of drug resistance. Substandard therapies include artemisinin monotherapies and products that contain inadequate or no artemisinin derivative [11,12].

In 2009, Phase 1 of the AMFm was launched with a $225 million commitment to support nine pilot programmes in eight countries (Cambodia, Ghana, Kenya, Madagascar, Niger, Nigeria, Tanzania, and Uganda). The Global Fund has established pricing guidelines for manufacturers participating in the AMFm, indicating the maximum price that manufacturers can offer to first-line buyers of ACT [13]. Countries participating in the AMFm may opt to set their own suggested retail prices (SRPs). Initial SRPs for an adult dose of ACT delivered to Phase 1 countries between August 2010 and April 2011 ranged from US $0.42 to US $1.00 [14].

An independent evaluation of the AMFm Phase 1 pilot programmes, conducted in March 2012 based on survey data collected between September 2009 and December 2010, found that AMFm QAACT was most widely available in public health facilities in all countries except for Nigeria and Madagascar [10]. Within all eight countries, the availability of QAACT in private outlets at baseline ranged from 6% in Niger to 27% in Nigeria. Non-artemisinin-based therapy was more commonly available than QAACT in all countries, and artemisinin monotherapy and low-quality ACT were more frequently available than QAACT in several countries [10].

Because patients receiving treatment in most public facilities and in many non-profit outlets do not pay for QAACT, the median cost of an adult dose was zero in public health facilities in all countries except for Ghana and in private non-profit outlets in Kenya, Madagascar, mainland Tanzania, and Uganda [10]. In private for-profit outlets, the median price for an adult dose ranged from $0.14 to $5.99. In private for-profit outlets, non-artemisinin anti-malarials were the least expensive treatments available in all countries, except Madagascar and Uganda [10].

The objective of this study was to assess anti-malarial stock and purchase patterns at private outlets in five AMFm Phase 1 countries in regard to three of the core AMFm goals: (1) increase the affordability of ACT; (2) increase the availability of ACT; and (3) crowd out monotherapies and other substandard therapies. All forms of ACT systematically assessed in this study have been evaluated by the WHO Prequalification of Medicines Programme.

## Methods

### Study population

Between April and May 2012, interviews were held in 598 private pharmaceutical outlets in five AMFm Phase 1 countries (Ghana, Kenya, Nigeria, Tanzania, and Uganda). Information regarding outlets that are primary recipients of AMFm products is available from several sources, including Novartis International AG, the Global Fund to Fight AIDS, Tuberculosis and Malaria, Population Services International, and in-country distributors. This information was compiled and compared to achieve an extensive sampling frame of outlets in each country that are primary recipients of AMFm products. From this list, approximately 80 outlets per country were selected using random sampling. In order to obtain a set of outlets that were not primary recipients of AMFm products, approximately 20 outlets per country not appearing on the AMFm recipient list were selected by convenience sampling. After examining geographic characteristics of the 100 outlets selected per country, it was determined that the majority were located in urban or semi-urban areas. In order to better understand the impact of AMFm in rural areas, an additional 20 rural outlets per country were selected. For the sampling of rural outlets, two districts per country were chosen at random from among all districts containing urban outlets that are primary recipients of AMFm products. From each of these districts, convenience and snowball sampling were used to select ten rural outlets, giving a total of 120 outlets per country in Ghana, Nigeria, and Tanzania, and 119 outlets per country in Kenya and Uganda. Of the 598 outlets included in this study, 434 (72.6%) were urban and 164 (27.4%) were rural.

### Data collection

Qualitative interviews were conducted by telephone with ten national (two per country) and five international malaria experts. The purpose of these interviews was to identify current trends and challenges in malaria control, AMFm effectiveness, and ACT access in the study countries. The results of these interviews were used to develop two questionnaires: 1) A quantitative questionnaire that gathered information on stock and sales for AMFm and non-AMFm versions of 10 QAACT products, top-selling anti-malarial and ACT products, major suppliers, factors influencing anti-malarial stock and recommendation decisions, rapid diagnostic testing, and perceptions around ACT purchase and use (Outlet Survey tool). Data were also collected on the licensure status of each outlet. Country regulations often stipulate that medication outlets must obtain and periodically renew a license for the importation, sale, dispensing, preparation, and compounding of both over the counter (OTC) drugs and prescription only medicines (POM). Local interviewers, who were trained in proper interview and data collection methodologies, administered the structured questionnaire to either the outlet manager or the most senior employee responsible for drug sales present at the time of the outlet visit. 2) An open-ended, semi-structured qualitative questionnaire that was used to assess the availability, use, and patient perceptions of both ACT overall and AMFm products (Qualitative Survey). This questionnaire also examined differences between urban and rural areas in regard to malaria treatment perceptions and practices as well as ACT availability and use. For each country, a representative from the local study team completed one questionnaire.

### Data analysis

Data from the Outlet Survey tool were analyzed using SAS/STAT (SAS System 9.1, SAS Institute, Cary, NC, USA). The size of urban and rural outlets for each country was estimated using the total tablets sold in the past week for the top three selling ACT products. Overall stock management was assessed by determining the proportion of QAACT products for which there had been at least one stock-out occurrence in the previous three months, without drawing a distinction between AMFm and non-AMFm versions. Within-country results from urban and rural outlets were compared using chi-squared tests for independence. The mean number of stock-out days across all forms of QAACT assessed was also calculated. Within-country differences between urban and rural outlets were determined using appropriate *t*-tests.

The mean price of a full adult course of treatment for AMFm products was determined for both rural and urban areas in each country. This analysis was performed both pooled (across all brands) and by brand. Additionally, the mean price of a full adult course of the top-selling ACT product, which included both QAACT and non-QAACT, was calculated. Within-country differences for urban and rural areas were determined for each mean price using appropriate *t*-tests. Results from the Qualitative Survey were analyzed for themes using HyperRESEARCH (HyperRESEARCH 3.0, ResearchWare, Randolph, MA).

## Results

### Outlet characteristics

Outlet characteristics are summarized in Table 1. Results are stratified by outlet location, either urban or rural. In urban Ghana, Kenya, Tanzania, and Uganda, the majority of the outlets surveyed were pharmacies or private clinics (87.0%, 98.6%, 92.7%, and 89.9% respectively). In Nigeria, only 13.4% of outlets were pharmacies or private clinics. With the exception of Kenya and Uganda, the majority of rural outlets surveyed were neither pharmacies nor private clinics but instead smaller drug shops or patent medicine stores. Patent medicine stores are licensed for the sale and distribution of OTC drugs only. In Uganda, 41.0% of rural outlets indicated that a pharmacy was their first line supplier, compared with only 8.1% of urban outlets. Similar trends were seen in Tanzania (see Additional file 1).

**Table 1:** Characteristics of urban and rural outlets selected in Ghana, Kenya, Nigeria, Tanzania, and Uganda

	**Ghana**		**Kenya**		**Nigeria**		**Tanzania**		**Uganda**	
	**Urban (n = 100)**	**Rural (n = 20)**	**Urban (n = 77)**	**Rural (n = 42)**	**Urban (n = 82)**	**Rural (n = 38)**	**Urban (n = 96)**	**Rural (n = 24)**	**Urban (n = 79)**	**Rural (n = 40)**
**Outlet type, n (%)**										
Pharmacy	77 (77.0)	-	76 (98.6)	40 (95.0)	8 (9.7)	2 (5.3)	81 (84.4)	2 (8.3)	31 (39.3)	2 (5.0)
Drug shop	13 (13.0)	20 (100.0)	-	-	11 (13.4)	2 (5.3)	3 (3.1)	8 (33.3)	5 (6.3)	18 (45.0)
Private clinic/Health facility	10 (10.0)	-	-	2 (5.0)	3 (3.7)	1 (2.6)	8 (8.3)	4 (16.7)	40 (50.6)	20 (50.0)
Patent medicine store	-	-	-	-	54 (65.9)	32 (84.2)	-	-	-	-
Other	-	-	1 (1.4)	-	6 (7.3)	1 (2.6)	4 (4.2)	10 (41.7)	3 (3.8)	-
**Outlet size, Median (Range)**										
Total tablets sold in the past week per outlet for the top 3 ACT products	50 (0–1860)	14 (0–380)	420 (0–13725)	411 (0–16800)	414 (0–532800)	408 (0–7070)	350 (0–212800)	360 (4–1296)	480 (0–74800)	234 (0–5040)
**Licensed (%)**	100.0	100.0	97.2	85.0	59.8	39.5	100.0	100.0	100.0	100.0

The total number of tablets for the three top-selling forms of ACT sold in the week prior to the study was used as a proxy for outlet size. As shown in Table 1, outlets surveyed in Ghana were smaller than outlets surveyed in the other four countries, especially in rural areas. Data were also gathered on the license status of each outlet, showing that fewer outlets surveyed in Nigeria (59.8% of urban and 39.5% of rural outlets) were licensed compared with outlets in the other four countries (100% for rural and urban outlets in Ghana, Tanzania, and Uganda; 97.2% and 85.0% of urban and rural outlets, respectively, in Kenya).

### ACT affordability

The AMFm is designed to reduce the final purchase price of QAACT. To examine price trends across products, a pooled price analysis was performed that included 1,343 prices for AMFm products and 239 prices for corresponding non-AMFm products. This analysis showed that the mean price of a full adult course of treatment varies widely between countries. The highest mean price was seen in urban Uganda (US$2.42) and the lowest in rural Kenya and Tanzania (US$0.72) (Table 2). Significant price variation was also observed within some countries between rural and urban outlets. In Nigeria, the mean price of AMFm products in rural areas was significantly higher than the mean price in urban areas (US$1.24 vs. US$0.98, p < 0.0001); this finding held true for the top-selling ACT product (US$1.40 vs. US$1.00, p = 0.0007). In Uganda, the opposite pattern was seen, where both the AMFm mean price and the top-selling ACT mean price were significantly higher in urban outlets than rural outlets (US$2.42 vs. US$1.85, p = 0.0065 for AMFm and US$2.40 vs. US$1.78, p = 0.0053 for top-selling ACT). Similarly, in Ghana the top-selling ACT product in urban outlets was significantly more expensive than the top-selling product in rural outlets (US$2.47 vs. US$0.88, p < 0.0001). However, unlike other countries and areas, the top-selling ACT product in Ghana was not also a product within the AMFm QAACT list.

**Table 2:** Average price of an adult dose of anti-malarial treatment in urban and rural outlets (US$)

	**Ghana**			**Kenya**			**Nigeria**			**Tanzania**			**Uganda**		
	**Urban (n=157)**	**Rural (n=31)**	**p**	**Urban (n=266)**	**Rural (n=127)**	**p**	**Urban (n=239)**	**Rural (n=110)**	**p**	**Urban (n=148)**	**Rural (n=40)**	**p**	**Urban (n=154)**	**Rural (n=73)**	**p**
**All AMFm products Mean (SD)**	1.14 (1.58)	0.85 (0.13)	0.3066	0.79 (0.40)	0.72 (0.23)	0.0717	0.98 (0.54)	1.24 (0.62)	< 0.0001	0.85 (0.94)	0.72 (0.20)	0.3971	2.42 (1.71)	1.85 (0.70)	0.0065
Top-selling ACT* Mean (SD)	2.47** (0.37)	0.88 (0.16)	< 0.0001	0.78 (0.29)	0.72 (0.24)	0.3078	1.00 (0.44)	1.40 (0.78)	0.0007	0.65 (0.14)	0.72 (0.20)	0.0929	2.40 (1.10)	1.78 (0.60)	0.0053
**Country-specific SRP**[14]	1.00			0.45			0.50			0.65			0.42		

*Prices given for the AMFm version of each product.

**Not an AMFm product.

In this study, the mean price of AMFm products exceeded the SRP in each country in all outlets except those in rural Ghana. The mean price of the top-selling ACT also exceeded country-specific SRPs for all outlets other than rural outlets in Ghana and urban outlets in Tanzania [14]. The greatest differential between SRP and actual prices occurred in Uganda, where the mean prices of AMFm and top-selling ACT products in rural (US$1.85 and US$1.78) and urban (US$2.42 and US$2.40) areas were approximately four to six times the SRP (US$0.42), respectively.

Despite final purchase prices that exceed SRPs, the 10 AMFm QAACT medications included in this analysis remain less expensive than non-AMFm versions of identical drugs. The mean price of a full adult course of AMFm Coartem (artemether-lumefantrine, Novartis, Switzerland), the most widely available QAACT of the 10 medications included in this analysis, ranged in urban outlets from US$0.82 (SD = 0.13) to US$2.39 (SD = 1.10). In contrast, the price of non-AMFm Coartem in these outlets ranged from US$6.12 (SD = 5.78) to US$9.32 (SD = 4.79). Results were similar in rural outlets, with prices ranging from US$0.69 (SD = 0.20) to US$2.06 (SD = 0.67) for AMFm Coartem and US$3.51 (SD = 3.82) to US$9.37 (SD = 6.50) for non-AMFm Coartem. Prices for Lumartem (artemether-lumefantrine, Cipla, India), with the second-highest availability in both urban and rural outlets, showed a similar price discrepancy. Mean AMFm prices in all outlets varied from US$0.82 (SD = 0.14) to US$2.28 (SD = 1.09) and non-AMFm prices from US$0.63 (SD = 0) to US$6.72 (SD = 3.43). Overall, full-price QAACT was found to be up to eight times more expensive than those subsidized through the AMFm.

Although AMFm ACT has lower purchase prices compared with their non-AMFm counterparts, qualitative findings indicate that affluent customers may prefer to purchase the more expensive version of an ACT due to perceptions that a less expensive drug is also less effective. Based on data from the Qualitative Survey, in three (Ghana, Nigeria, and Tanzania) of the five study countries there is a shared perception that AMFm-subsidized artemether-lumefantrine is less effective than the unsubsidized version of the same brand. The drastic difference in price between AMFm subsidized and unsubsidized artemether-lumefantrine may lead some individuals to believe that a reduction in price is inherently coupled with a reduction in quality. In Ghana, where unsubsidized ACT has been on the market for a long period of time, those who can afford the non-AMFm version of the brand often purchase it because of the perception that the non-AMFm version is both safer and more effective. Similar opinions were found in Nigeria and Tanzania. Additionally, data from the Qualitative Survey revealed that consumers strongly prefer fewer tablets per dose, a factor which is taken into account when selecting and adhering to an anti-malarial treatment course.

### ACT availability

Outlets were surveyed for the availability of anti-malarial medications, specifically those included on the list of QAACT with both an AMFm and non-AMFm version (Arsuamoon, Artefan, Artemef, artemether-lumefantrine, artesunate-amodiaquine, Coartem, CoFalcinum, Combusinate, Falcimon Kit, and Lumartem). In urban areas, the proportion of outlets that had at least one AMFm QAACT in stock at the time of visit ranged from 75.3% in Kenya to 96.3% in Nigeria. In rural areas this figure varied from 64.3% in Kenya to 95% in Ghana.

At each outlet, survey respondents were asked to indicate if one or more of these forms of QAACT, determined to be normally in stock, had been out of stock in the previous three months (Table 3). This analysis did not distinguish between AMFm and non-AMFm QAACT. In Ghana and Kenya, there was a significantly higher percentage of stock-out occurrences in rural areas compared with urban areas (26.8% vs. 54.6%, p = 0.0013 in Ghana and 78.4% vs. 87.0%, p = 0.0237 in Kenya). The mean duration of stock-out across all products varied among countries, with the longest time occurring in rural Kenya, 48.0 days (Standard Deviation [SD] = 23.1), and the shortest in rural Ghana, 13.3 days (SD = 7.9). While the durations of stock-outs differed between countries, they were consistent between urban and rural outlets in each country, with the exception of Ghana. In Ghana, stock-out duration was significantly shorter in rural areas than urban areas (p = 0.0120). Outlets in Nigeria were not included in this analysis due to data collection assumptions for this stock variable that differed from the other four study countries.

**Table 3:** Stock management of QAACT in urban and rural outlets*

	**Ghana**			**Kenya**			**Tanzania**			**Uganda**		
	**Urban (n = 213)**	**Rural (n = 33)**	**p**	**Urban (n = 338)**	**Rural (n = 154)**	**p**	**Urban (n = 170)**	**Rural (n = 49)**	**p**	**Urban (n = 186)**	**Rural (n = 77)**	**p**
**QAACT with at least one stock-out occurrence in the last 3 months (%)**	26.8	54.6	0.0013	78.4	87.0	0.0237	20.6	20.4	0.9781	31.7	33.8	0.7469
**Total days out of stock Mean (SD, Median)**	32.4 (31.0, 21.0)	13.3 (7.9, 14.0)	0.0120	43.6 (24.6, 40.0)	48.0 (23.1, 60.0)	0.6796	26.4 (20.7, 28.0)	21.0 (16.9, 23.0)	0.4543	28.1 (25.3, 21.0)	28.8 (30.0, 21.0)	0.9132

*Data not available for Nigeria.

**Figure 1:**
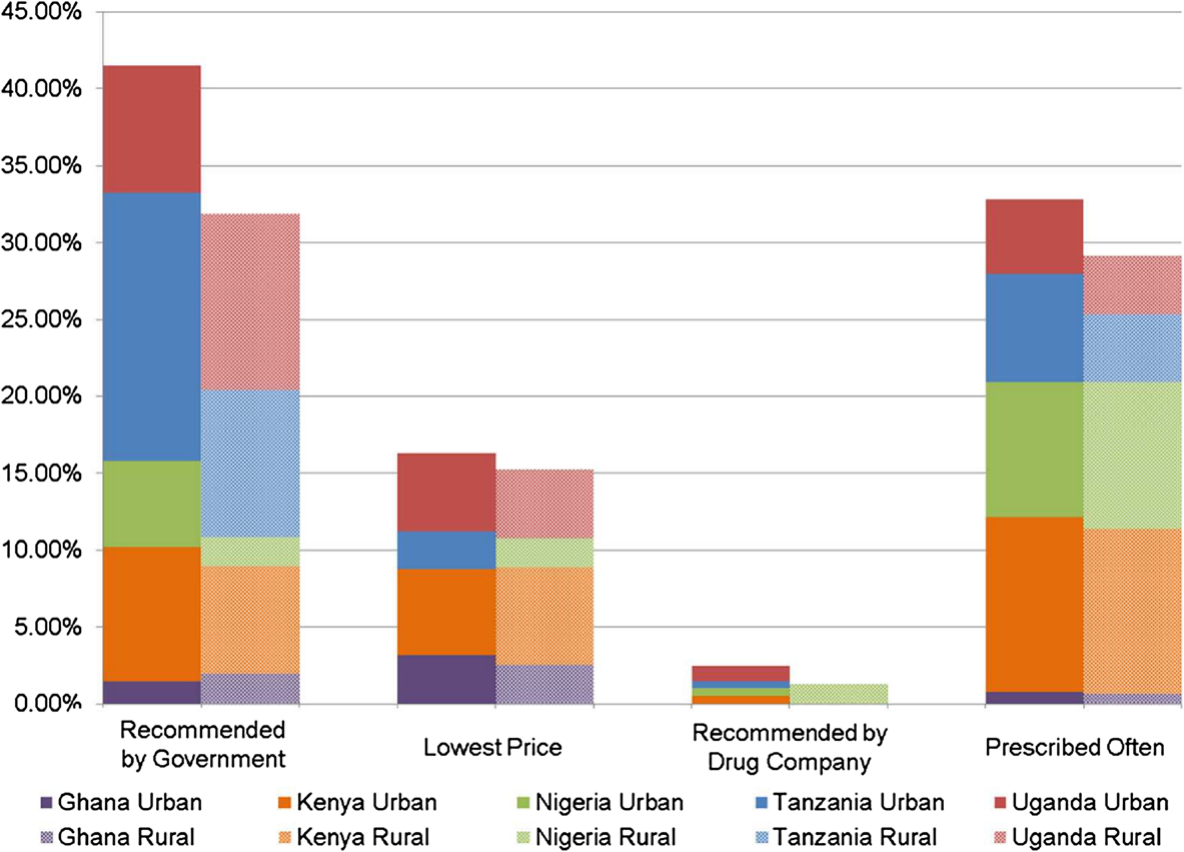
Factors that affect anti-malarial stock decisions: pooled analysis, urban (n = 412) and rural (n = 158).

A pooled analysis of all countries was undertaken to identify the extent to which factors such as government and drug company recommendations (including marketing), medication price, and physician prescription patterns impact anti-malarial stock decisions. In this analysis, government recommendation was the most significant factor influencing anti-malarial stock choices, cited by 41.5% and 31.9% of urban and rural outlets, respectively (Figure 1). The frequency with which specific anti-malarials are prescribed also plays a role, especially in Kenya and Nigeria. In Kenya and Uganda, final patient price impacts stock decisions. However, drug manufacturer recommendations had little to no effect on the choice of anti-malarials stocked in any country.

### Crowd-out of monotherapies

A key objective of the AMFm programme is to “crowd out” artemisinin monotherapies and other substandard therapies. In order to evaluate the success of this aspect of the programme, outlets were surveyed to determine their three top-selling anti-malarial drugs (Table 4). The three top-selling anti-malarials reported for both urban and rural areas in each country were, with the exception of rural Uganda and urban Nigeria, combination therapies. In rural Uganda, quinine was reported as the second top-selling anti-malarial drug. In urban Nigeria, artesunate monotherapy was listed as the second top-selling anti-malarial. When outlet managers in each country were asked which anti-malarial they would recommend for a case of uncomplicated malaria in either adults or children, there were instances of monotherapy recommendations in all study countries except Kenya.

**Table 4:** Three top-selling anti-malarial products by volume for urban and rural outlets

	**Ghana**		**Kenya**		**Nigeria**		**Tanzania**		**Uganda**	
	**Urban (n = 100)**	**Rural (n = 20)**	**Urban (n = 77)**	**Rural (n = 42)**	**Urban (n = 82)**	**Rural (n = 38)**	**Urban (n = 96)**	**Rural (n = 24)**	**Urban (n = 79)**	**Rural (n = 40)**
**Top-selling**	Coartem	Lumartem	Coartem	Coartem	Coartem	Coartem	Coartem	Orodar	Coartem	Lumartem
**Second top-selling**	Lonart	Lonart	Duo Cotecxin	Artefan	Artesunate (Generic)	Lumartem	Duo Cotecxin	Lumartem	Duo Cotecxin	Quinine (Generic)
**Third top-selling**	Lumartem	Alaxin	Alaxin	Artemether- Lumefantrine (Generic)	Arsuamoon	Arsuamoon	Metakelfin	Coartem	Lonart	Coartem

## Discussion

The results of this study highlight the important position held by QAACT, specifically AMFm QAACT, within the private sector. AMFm-subsidized QAACT products are less expensive than their non-AMFm counterparts. Results from this study are consistent with a recent price survey for AMFm and non-AMFm artemether-lumefantrine in six African countries [14]. However, the current study shows that in countries where AMFm drugs may be perceived as less effective, wealthier patients will pay the additional cost for non-AMFm medications. Further research will be needed to explore the scale of this perception and behavior. Mitigating the negative perceptions of subsidized drugs will also be important for increasing use of QAACT in the WHO Africa region. Potential mitigation activities include social marketing and education focused on the message that lower prices are not necessarily synonymous with lower quality. In addition to consumer perceptions of the AMFm, qualitative interviews revealed that consumers strongly consider the number of tablets per dose when selecting an ACT. Consequently, reducing the number of tablets in each dose will be critical for increasing the use of QAACT.

The AMFm has resulted in a lower final purchase price for a number of ACT products. Despite this success, the price of AMFm anti-malarials reported in this study exceeds the SRPs in all outlets other than those in rural Ghana. Additional evaluation of factors driving stocking decisions among outlets and purchasing decisions among consumers will be important for the achievement of prices closer to SRPs. Given that price remains a factor in the type of anti-malarial recommended in some areas, further reduction in the price of QAACT is important to achieving crowd-out of artemisinin monotherapies and substandard anti-malarial regimens. Additionally, given that government recommendation was the most significant factor in stocking decisions, it is critical that governments remove artemisinin monotherapies and substandard anti-malarial treatments from national and institutional treatment guidelines and discontinue the regulatory approvals of these drugs.

Findings from this study also emphasize that although there are within- and between- country differences in AMFm QAACT availability, overall market penetration of one or more AMFm QAACT products is high, even in rural areas. This finding is in contrast to other survey data gathered between 2009 and 2010, and suggests that QAACT availability in the private sector may be improving over time [10].

This study also highlights key differences between the types of outlets that sell anti-malarials in urban and rural areas, which has implications for procurement and supply chain management of anti-malarial stocks. In Uganda and Tanzania, rural outlets, in comparison with urban outlets, reported that they more frequently purchase anti-malarials from pharmacies. These pharmacies, in turn, are supplied directly by drug wholesalers. While this study did not evaluate the complete supply chain of each outlet, this finding suggests that efforts to include outlet types such as drug shops and patent medicine stores in the primary distribution strategy may help to increase access to, and affordability of, QAACTs in rural areas. Further evaluation of anti-malarial supply chains is needed to explain the significantly higher occurrence of stock-outs in rural versus urban outlets in Ghana and Kenya. Supply shortages related to procurement difficulties have been shown to affect the cost of ACT in Cambodia, another AMFm Phase 1 country [15]. Additional insight is also needed regarding the impact of product diversion on QAACT affordability and availability.

The continued popularity of monotherapies indicates that more work needs to be done toward achieving the crowd-out goal of the AMFm. For example, in rural Uganda, 41% of outlets indicated that they consider cost when making recommendations to customers, and in this setting monotherapies may still be the least expensive anti-malarial available. This could account for the appearance of quinine as the second top-selling anti-malarial in rural Uganda.

Despite this result and the finding that artesunate monotherapy was the second top-selling anti-malarial in urban Nigeria, the overall results of this study with respect to crowd-out appear to show improvement compared with previous assessments. With the exception of rural Tanzania, the top-selling anti-malarial product in all countries, in both urban and rural areas, was a QAACT. In contrast, data collected in 2009 and 2010 found that the anti-malarials with the highest market share were non-artemisinin therapies in all countries surveyed, including Ghana, Kenya, Nigeria, Tanzania, and Uganda [10]. Based on evidence from the current study, a direct causal argument cannot be drawn between the introduction of the AMFm and subsequent QAACT uptake. Still, it is likely that the AMFm acted as a catalyst for the improved accessibility of all QAACT products through both increased supply-side pressure and augmented supply channels. The expansion of country-specific awareness programmes focused on the benefits of QAACT and the risks of substandard therapies will help to ensure a more efficient and effective crowd-out process.

Following the end of the pilot phase, it was decided that AMFm would be discontinued and the AMFm model integrated into routine Global Fund grants [16]. There were a number of factors leading to this decision, including concerns about the sale of anti-malarial drugs to febrile patients who are not *P. falciparum*-positive and minimal private sector uptake for paediatric formulations [17]. While efforts to increase diagnostic testing, appropriate use of anti-malarial drugs, and uptake of paediatric treatments are warranted, these objectives would benefit from continued efforts to improve QAACT availability and affordability. In the absence of AMFm subsidies, new strategies will be needed to ensure continued use of QAACT.

The findings presented here support the introduction of programmes similar to AMFm as important mechanisms for transforming malaria treatment and driving the use of QAACT in place of artemisinin monotherapy and other substandard therapies. Results from this study complement a recent AMFm Phase 1 evaluation, which found substantial increases in QAACT availability (25.8-51.9 percentage points) and market share (15.9-40.3 percentage points) in Ghana, Kenya, Nigeria, Tanzania, and Uganda coupled with a decrease in final QAACT purchase price for each country except Uganda [18].

### Limitations

A key study limitation included the fact that information related to customer and retailer treatment choice and retailer ACT recommendations was based on responses from outlet salespeople rather than direct observation. In some cases, outlet personnel may have provided inaccurate information, either unintentionally due to incomplete knowledge of outlet practices, or intentionally in order to present information perceived as desirable. To address this limitation, qualitative findings were used to supplement and aid in the interpretation of the quantitative data. Additionally, this assessment is limited by the absence of data on the occurrence and duration of stock-outs in Nigeria, which arose from differences in data collection assumptions between Nigeria and the other participating countries. Despite these limitations, this study provides new insights regarding the impact of the AMFm on QAACT availability, QAACT affordability, and crowd-out of monotherapies in five Phase 1 countries.

## Conclusions

Mean AMFm prices in all countries were found to exceed SRPs, potentially hindering the broader uptake of QAACT. Additionally, monotherapies continue to be dispensed and used in both urban and rural outlets. Still, overall AMFm QAACT availability was high, even with stock-out and supply chain discrepancies between urban and rural areas. Continued efforts to improve QAACT affordability and accessibility could play a crucial role in reducing malaria mortality and drug resistance in endemic countries, especially if combined with initiatives such as increased deployment of rapid diagnostic testing, frequent supply chain monitoring, and retailer and customer education programmes.

## Competing interests

This study was supported by an unrestricted grant from Novartis International AG.

## Authors’ contributions

All authors participated in study conception and design. BD, KS, and ET participated in data acquisition and extraction. BD, JL, KS, and JS performed statistical analysis, interpretation, drafting, and critical revision. All authors read and approved the final manuscript.

## Supplementary Material

Additional file 1 **“Table: Primary suppliers of anti-malarial medication for urban and rural outlets”.** Personnel at each outlet were asked to indicate the primary supplier of anti-malarial medications. This table indicates the number and percentage of outlets citing each supplier type as their primary source of anti-malarials. Click here for file
